# Characteristics of upper respiratory tract rhinovirus in children with allergic rhinitis and its role in disease severity

**DOI:** 10.1128/spectrum.03853-23

**Published:** 2024-05-23

**Authors:** Yingqin Gao, Jienan Zhou, Meilan Wang, Yanqi Liang, Ting Zhang, Yunxiang Mao, Jing Ma, Li Li, Tiesong Zhang, Lei Guo

**Affiliations:** 1 Affiliated Children’s Hospital of Kunming Medical University, Kunming, China; 2 Yunnan Center for Disease Control and Prevention, Kunming, China; 3 Dali University, Dali, China; Quest Diagnostics, Secaucus, New Jersey, USA

**Keywords:** allergic rhinitis, rhinovirus, genotype, disease severity, children

## Abstract

Allergic rhinitis (AR) is a global health challenge that particularly affects the quality of life of children. Human rhinovirus (HRV) infection usually causes common cold in the upper respiratory tract (URT) and can also affect airway allergy development, such as asthma exacerbation, but its relationship with AR is poorly understood. The study aimed to gain insight into the characteristics of HRV that is prevalent in AR children and its role in AR severity. A total of 362 children with symptomatic AR were enrolled from southwestern China during 2022–2023, and nasal lavage samples were collected for HRV molecular characterization and cytokine measurement. HRV was detected in 40% of the AR children, with peak detection in autumn. The positive rate was not correlated with whether the subjects were under allergen-specific immunotherapy (AIT). Among the detected HRVs, 42% were species A, 36% were species B, and 22% were species C, involving 21 A genotypes, 6 B genotypes, and 7 C genotypes. HRV positivity was significantly associated with symptom severity (visual analog scale [VAS] score) and elevated levels of local nasal IgE, interleukin-25 (IL-25), IL-4, and CXCL13 in AR children who did not receive antiallergic treatment. All three species of HRV strains (A1B, A21, B27, B70, and C17) had been isolated and were able to infect respiratory epithelial tissue *in vitro*. Complete genome sequencing showed that the antigenic epitopes of the isolated HRVs had certain variations. Our work reveals the etiological characteristics of URT-HRV in AR children and suggests a role of HRV infection in the pathogenesis of childhood AR.

**IMPORTANCE:**

Our study revealed high human rhinovirus (HRV) detection rate in children with allergic rhinitis (AR), and HRV infection (A, B, or C species) is positively associated with the symptom severity in AR children. Elevated nasal IgE, interleukin-25 (IL-25), IL-4, and CXCL13 levels suggest a potential pathogenic mechanism by which HRV infection induces nasal type 2 immune/inflammation responses and local IgE production in AR patients. In addition, etiological analysis found that the main prevalent HRV species in AR children are A and B (~80%), which is different from acute respiratory infection and asthma exacerbation, where species A and C are dominant. The data reveal the distinct species prevalence characteristics of HRV infection in AR. Finally, we isolated all three species of HRV strains from nasal cavity of AR children with varying degrees of antigenic epitope mutations and in vitro infectivity, highlighting the importance of strengthening monitoring and intervention for respiratory HRV infection in AR children.

## INTRODUCTION

Allergic rhinitis (AR) is a common non-infectious inflammatory disease of the nasal mucosa caused by allergens, which affects approximately 19.1% of the global population (1). AR can occur at all ages, with a high incidence in children and adolescents. The prevalence rate of AR has shown a rapid upward trend in recent years, especially in China, which has substantially affected the learning and quality of life of patients (2, 3). Besides, AR is usually clinically relevant and can be complicated by multiple chronic respiratory diseases, including chronic sinusitis, conjunctivitis, and asthma. As “unified airway diseases,” AR and asthma are often accompanied by one another. It is estimated that up to 80% of patients with asthma are affected by AR, while up to 40% of AR patients also have asthma (4). Uncontrolled AR can lead to exacerbation of combined AR and asthma syndrome (5).

Human rhinovirus (HRV) belongs to the *Picornaviridae* family and is divided into three species: A, B, and C. Multiple genotypes are a major feature of the virus. According to the Picornaviridae Study Group Subcommittee website, there are 169 rhinovirus genotypes to date, including 80 genotypes A, 32 genotypes B, and 57 genotypes C. HRV mainly infects the upper respiratory tract (URT), causing viral rhinitis and sinusitis. Studies show that HRV can also invade the lower respiratory tract (LRT), causing bronchitis, severe pneumonia, chronic obstructive pulmonary disease (COPD), and asthma acute attack or exacerbation (6). Currently, knowledge about the relationship between HRV infection and airway allergy is mainly focused on asthma, and the virus plays a crucial role in asthma exacerbation (7). Few studies have reported the relationship between HRV infection and URT allergies, such as AR. Previous studies in both adults and children have revealed that AR is associated with higher levels of URT virus infection compared to non-allergic subjects, with HRV being the dominant virus (8, 9), but there is no significant association between HRV detection and disease severity in AR children (9). However, given that the study (9) enrolled children who took antihistamines to relieve symptoms, this result may be unreliable. Whether HRV infection contributes to AR aggravation has not been elucidated. Therefore, this study investigated and analyzed the characteristics of URT-HRV in AR children and evaluated the correlation between HRV positivity and disease severity of AR, which will benefit the understanding of HRV infection in the pathogenesis of AR and respiratory allergies.

## MATERIALS AND METHODS

### Patients and clinical evaluation

**Fig 1:**
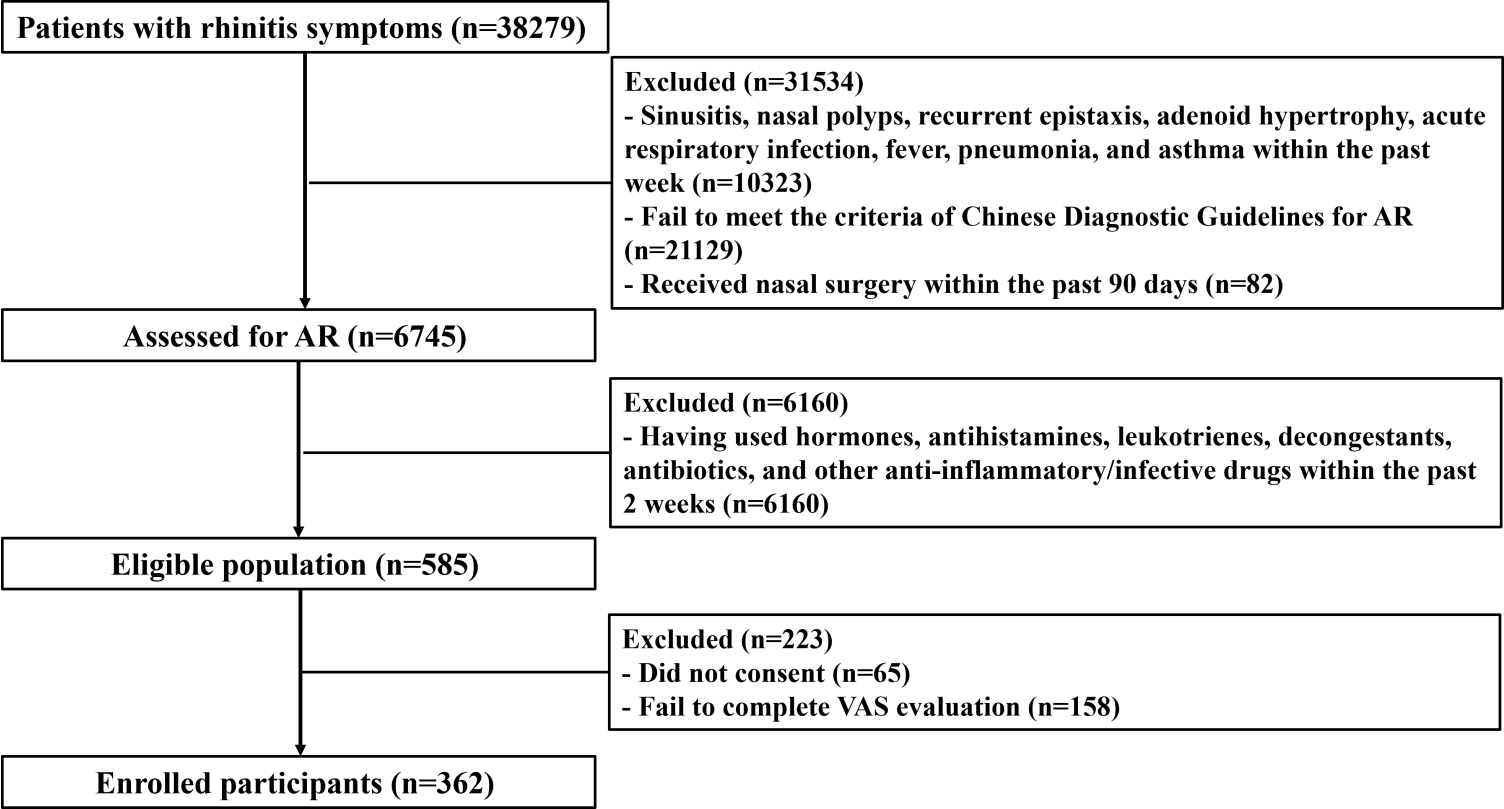
Flow diagram of study screening and eligibility.

Patients who visited the Department of Otolaryngology Head and Neck Surgery of Kunming Children’s Hospital due to symptoms of rhinitis underwent enrollment screening from April 2022 to April 2023 (Fig. 1). AR diagnostic criteria are in accordance with the Chinese Guidelines for the Diagnosis and Treatment of Allergic Rhinitis (2022, revised version) (10). The diagnosed children must meet the requirements of at least one allergen skin prick test and/or serum-specific IgE positivity or nasal provocation test positivity, in addition to the symptoms of typical AR, such as persistent paroxysmal sneezing, clear nasal discharge, nasal itching, and nasal congestion. The exclusion criteria for the experimental group are shown in Fig. 1. The enrolled patients, together with their guardians, filled out a visual analog scale (VAS) to assess the severity of allergic diseases (11). The patient should mark the corresponding level of disease perception on a scale of 0–10, with 0 being asymptomatic or troubleless and 10 being very serious or extremely troubled. The research was reviewed by the hospital ethics committee (ethical approval number 2022-03-056-K01), complies with relevant regulations, and was conducted with the informed consent of the child’s guardian.

### HRV detection

Nasal lavage fluids (saline washing) were collected from the enrolled AR children. The subject extends their neck horizontally by approximately 30° while sitting. Five milliliters of normal saline (0.9%) were injected into both nasal cavities using a disposable sterile syringe while the subject is not breathing or swallowing. After 10 seconds, the subject bent their neck and forcefully discharged the mixture of mucus and saline into a sterile collection container (collection volume 5–6.5 mL). Immediately after collection, centrifuge the lavage fluid at 12,000 × *g* for 10 minutes at 4°C to collect the supernatant. The sample was aliquoted and frozen at −80°C until analysis was conducted. Viral RNAs were extracted from the lavage samples using the MiniBEST Viral RNA/DNA Extraction Kit (TaKaRa Biotechnology, Cat. 9766), and the nucleic acid concentration and purity were determined using the NanoDrop One Spectrophotometer (Thermo Fisher). The HRV-specific PCR primers and probe were synthesized by Beijing Qinke Biological Company as previously reported (12). Viral nucleic acids were identified by real-time PCR (RT-PCR) (Ct <34）with a One Step PrimeScript RT-PCR Kit (Perfect Real Time) (TaKaRa, Cat. RR064). Viral positive samples were further amplified by reverse transcription PCR for the viral genome VP4/VP2 sequence (13), and the amplified fragments were sequenced and blast compared with the corresponding virus strains in GenBank to identify different genotypes of HRV.

### Virus isolation

One hundred fifty microliters of nasal lavage fluid samples was inoculated onto H1-HeLa cells (ATCC-CRL-1958) in a 24-well culture plate and continuously cultured in an incubator at 35°C and 5% CO_2_ for 7 days, and cytopathic effects (CPEs) were observed after 3 days. The cells were blindly passaged for three consecutive passages, and no further cell experiments were performed without CPE or negative nucleic acid tests.

Nasal mucosa cells from the inferior turbinate of the healthy researchers (*n* = 3, age range 30–40, man to woman 1:2) were scraped and collected. The cells were expanded and cultured with PneumaCult-Ex Plus Medium (STEMCELL, Cat. 05040) and differentiated into respiratory epithelial tissue organoids by air–liquid interface (ALI) culture with PneumaCult-ALI Medium (STEMCELL, Cat. 05001) on Transwell cell culture dishes (12-mm diameter inserts, 0.4-µm pore polyester membrane, Costar, Cat. 3460) (14). Two hundred microliters of nasal lavage fluid collected from each subject was added to the ALI-cultured respiratory epithelial cells in the Transwell inserts and incubated for 1.5 hours, and then the liquid was aspirated and cultured for 7 days. The viral samples were blindly passaged for two consecutive passages, and no further cell experiments were performed if no nucleic acid test is positive.

### Complete genome sequencing

Viral RNA extracted from the virus stock was reverse transcribed into double-stranded cDNA (ds cDNA) using the Maxima H Minus Double Stranded cDNA Synthesis Kit (Thermo Fisher, Cat. K2561), and residual RNA was removed by adding RNase I. ds cDNA was purified by AMPure XP Magnetic Beads (Beckman, Cat. A63882), and purified ds cDNA was quantified by Qubit 3.0 Fluorescence Quantifier (Thermo Fisher). One nanogram of quantified ds cDNA was used to construct a sequencing library using a Nextera XT DNA Sample Prep Kit (Illumina, Cat. FC-131-1024/1096), and a Miseq Sequencing Kit v2 with 300 cycles (Illumina, Cat. MS-102–2002) was used for sequencing. The reference HRV strain sequences were downloaded from the National Center for Biotechnology Information (NCBI), and data were aligned and assembled using CLC Genomics Workbench Version 21 software (QIAGEN) to generate consensus sequences.

### Sequence alignment analysis

HRV reference sequences were downloaded from NCBI, and sequence alignment was performed using MEGA7.0 software. Molecular evolution analysis was performed by the neighbor-joining method (1,000 bootstrap operations), and phylogenetic trees were constructed. Nucleotide and amino acid homology and variation analyses were performed using CLC sequence view 8.0 software.

### Viral load and immunofluorescence

Viral nucleic acid extraction and RT-PCR detection were performed as described earlier. The VP2 gene of HRV was cloned into the pMD18-T vector, which was used to create a standard curve by 10-fold serial dilution. Viral copy numbers were normalized to the mass of RNA extracted from the original tissue samples and calculated based on the standard curve.

H1-HeLa cells or ALI cultured cells were infected with different HRV subtype viruses for 48–72 hours. Cells were then treated with 4% paraformaldehyde and 0.2% Triton X-100, blocked with 5% bovine serum albumin (BSA) for 1 hour at room temperature, and then incubated with anti-HRV VP3 antibody (Invitrogen, Cat. MA5-18250) (in ALI culture together with anti-zona occludens 1 (ZO-1) antibody, Proteintech, Cat. CL488-21773) overnight at 4°C. The next day, the cells were incubated with Alexa Fluor 488-conjugated goat anti-mouse IgG (Abcam, Cat. ab150113) or Alexa Fluor 647-conjugated goat anti-mouse IgG (Abcam, Cat. ab150115) for 1 hour at room temperature. Nuclei were counterstained with Hoechst 33342 (ProLong Glass Antifade Mountant with NucBlue, Invitrogen). Images were obtained with an LSM900 laser confocal microscope (Zeiss).

### Enzyme-linked immunosorbent assay

The levels of interleukin-4 (IL-4), IL-5, and IL-13 in the nasal lavage fluid were measured by enzyme-linked immunosorbent assay (ELISA) kits (Proteintech, Cat. KE00016, KE00217, and KE00020). The levels of IL-25, IL-33, and CXCL13 were tested by ELISA kits (Abcam, Cat. ab272200, ab223865, and ab269370). All assays were conducted according to the manufacturer’s protocols. The results were expressed as optical density (OD) at 450 nm and calculated according to the OD of the standard. IgE concentration was measured by ImmunoCAP (Thermo Fisher Scientific).

### Statistical analyses

Data are expressed as the mean ± standard deviation (SD) unless specified otherwise. Continuous variables are tested using two independent samples *t*-tests or Mann–Whitney *U* tests based on whether they conform to a normal distribution (Kolmogorov–Smirnov test). Fisher’s exact tests were used to compare categorical variables between groups. Determining the impact of various allergens on HRV-positive detection in AR children was performed using multivariable logistic regression analysis. Statistical analysis was performed with GraphPad Prism 9.0 software or Microsoft Excel software (Office 365), and the *P* value was calculated. *P* < 0.05 was considered statistically significant.

## RESULTS

### Prevalence and genotyping of HRV in AR children

A total of 362 AR children (mean age 3.5 years, range 0.6–12), who met the inclusion criteria, were enrolled in our 1-year study. The number of enrolled cases per month ranges from 24 to 42. The ratio of men to women was 3:2. Participants were from three provinces in southwestern China: Yunnan (68%), Guizhou (22%), and Sichuan (10%). The allergens of the subjects included one or more of the following: dust mites (*Dermatophagoides farinae* and *Dermatophagoides pteronyssinus*), animal dander, pollen, mold, eggs, and dairy products (Table 1). To maximize the sample size, the participants included patients undergoing allergen-specific immunotherapy (AIT) (52.21%, 189/362), including sublingual immunotherapy (mostly) and subcutaneous immunotherapy, with treatment periods ranging from 1 month to 3 years.

**TABLE 1:** Age, gender, and allergen distribution of HRV-positive/negative AR children^*a*^

Parameter	Total *N* = 362	HRV positives *n* = 145	HRV negatives *n* = 217	*P* value
Age (month) (median, interquartile range)	42 (25–70)	36 (24–68)	46 (27–71)	0.170
Men (%)	206 (57)	75 (52)	131 (60)	0.106
Allergens (%)				0.128
Pollen	138 (38)	60 (41)	78 (36)	0.381
Dust mite	169 (47)	61 (42)	108 (50)	0.458
Animal dander	61 (17)	22 (15)	39 (18)	0.726
Mold	24 (7)	13 (9)	11 (5)	0.225
Eggs	28 (8)	11 (8)	17 (8)	0.893
Dairy products	30 (8)	14 (10)	16 (7)	0.522

^*a*^ The intergroup statistical analysis was conducted using Mann–Whitney *U* test (age), Fisher’s exact test (gender), and multivariable logistic regression analysis (allergens). *P* < 0.05 was considered statistically significant.

**Fig 2:**
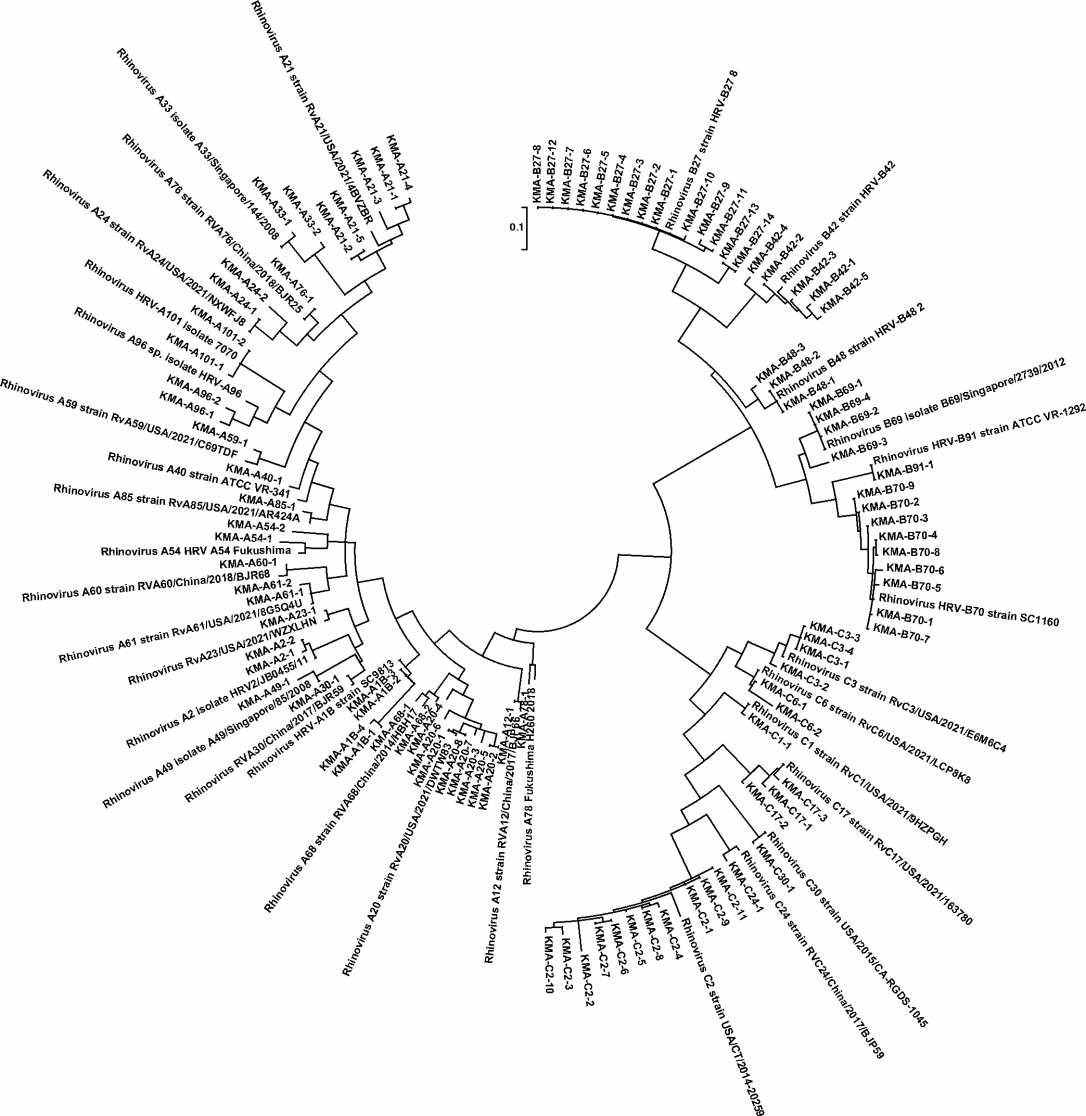
Phylogenetic analysis of the partial VP4/VP2 gene sequences of the 101 HRVs obtained in the study and reference HRV gene sequences from the GenBank database. Phylogenetic trees were constructed using the neighbor-joining method with 1,000 bootstrap replicates (MEGA, version 7.0). Sequences from the study were labeled as "KMA-HRV-genotype-serial number."

**Fig 3:**
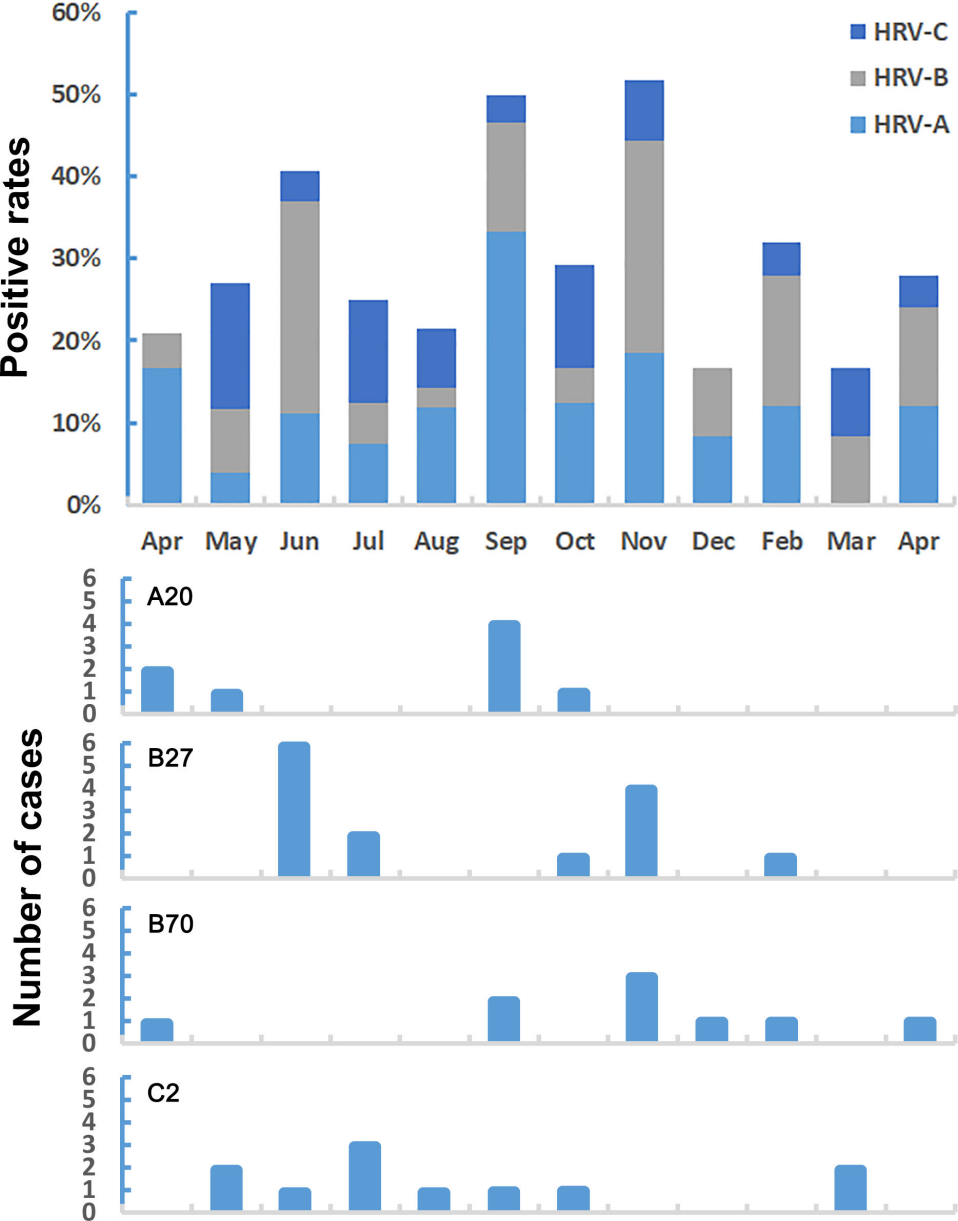
Monthly detection of different species and genotypes of HRV from 2022 to 2023.

HRV was detected in nasal lavage samples from 145 subjects, with a positive rate of 40.06%. Six of them were simultaneously detected with other common respiratory viruses, including adenovirus (three), influenza A virus (two), and respiratory syncytial virus (one). No significant differences were found in age, sex, and allergens between HRV-positive and HRV-negative AR children. (Table 1). Additionally, the HRV detection rate was 39.68% (75/189) in AIT children and 40.46% (70/173) in non-AIT children, indicating that HRV positivity was not related to whether AR children were treated with AIT (*P* = 0.915, Fisher’s exact test). A total of 145 HRV-positive samples were subjected to viral VP4/VP2 gene amplification, of which 101 were successfully amplified and sequenced. By aligning with HRV sequences in the GenBank database, 101 samples contained 42 species A, 36 species B, and 23 species C. A phylogenetic tree of the 101 rhinovirus sequences and their most homologous HRV strains in the GenBank database alignment was constructed (Fig. 2), and 21 genotypes A (A1, A2, A12, A20, A21, A23, A24, A30, A33, A40, A49, A54, A59, A60, A61, A68, A76, A78, A85, A96, and A101), six genotypes B (B27, B42, B48, B69, B70, and B91) and seven genotypes C (C1, C2, C3, C6, C17, C24, and C30) were identified. Among them, A20 accounted for 19.05% (8/42) of HRV-A, and B27 and B70 accounted for 38.89% (14/36) and 13.89% (9/36) of HRV-B, respectively. C2 had the highest proportion in HRV-C (47.83%, 11/23). Analysis of the detection rate of HRV in each month found that all three viral species can be detected throughout the year, with the peak detection occurring in autumn (September to November, up to 50%) (Fig. 3). The four major genotypes detected in each HRV species were not detected in specific months or clusters (Fig. 3).

### Disease severity and HRV detection

**Fig 4:**
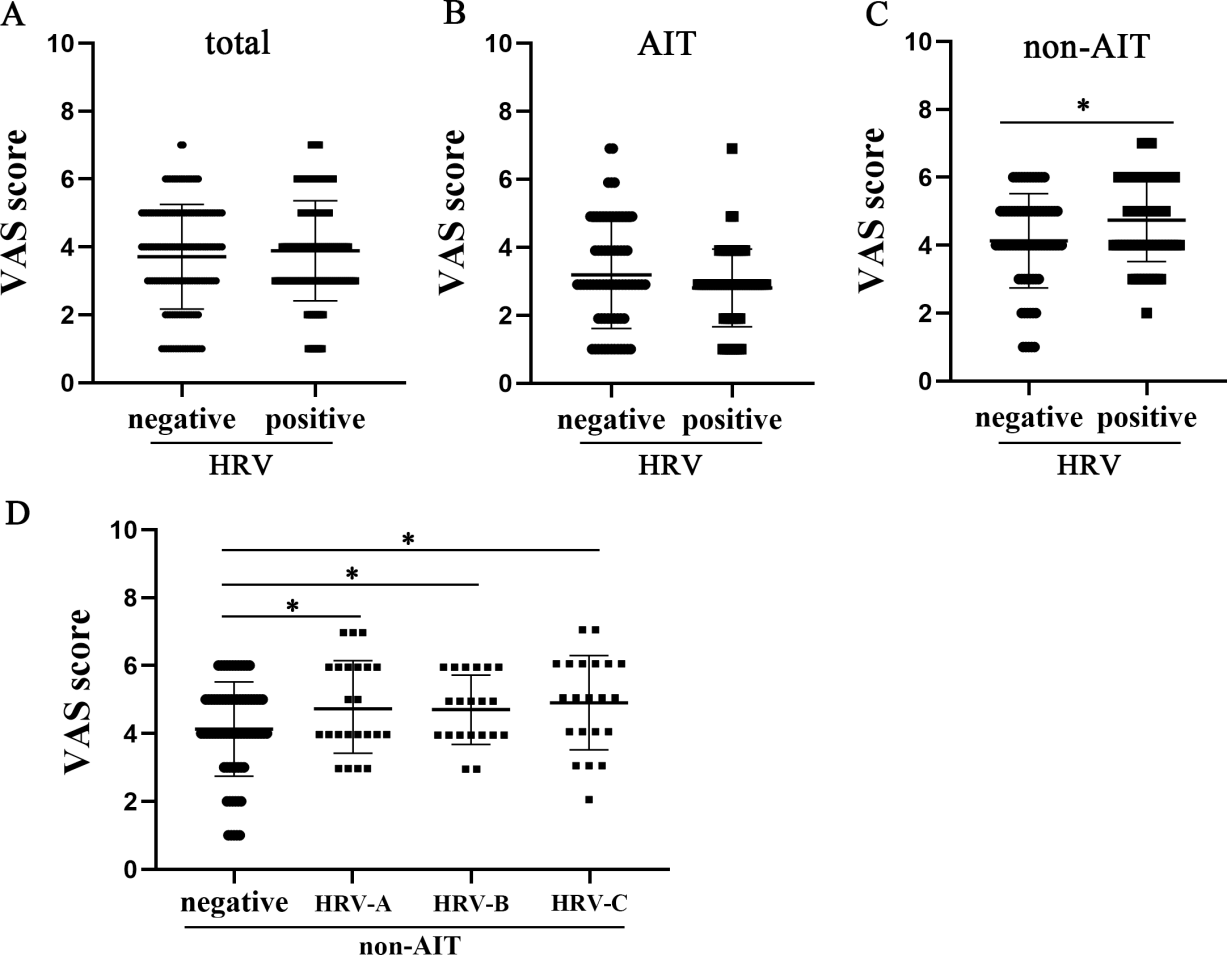
HRV detection and VAS scores of AR children. (**A**) VAS scores in HRV-positive (*n* = 145) and HRV-negative (*n* = 217) detection groups of AR subjects. (**B**) VAS scores in HRV-positive (*n* = 75) and HRV-negative (*n* = 114) detection groups of AR subjects under AIT treatment. (**C**) VAS scores in HRV-positive (*n* = 70) and HRV-negative (*n* = 103) detection groups of AR subjects without AIT treatment. (**D**) VAS scores in HRV-A (*n* = 23)-, HRV-B (*n* = 20)-, and HRV-C (*n* = 21)-positive detection groups compared with HRV-negative group (*n* = 103) in non-AIT AR subjects. Data are presented as the mean ± SD. ^*^P < 0.05 based on Mann–Whitney *U* tests (two-tailed for A–C, one-tailed for D).

The VAS rating of the severity of AR symptoms showed that the average score of subjects in the HRV-positive group was 3.89 ± 1.48, while the score of subjects in the negative group was 3.71 ± 1.54. The symptoms of both groups were concentrated in the mild to moderate VAS rating range, with no significant difference (Fig. 4A). Considering that the subjects included children under AIT treatment, we further divided the subjects into an AIT group (189) and a non-AIT group (173) and compared the correlation between HRV positivity and VAS scores in the two groups. In the AIT group, the VAS scores of the HRV-positive (75) and HRV-negative (114) subgroups were 2.90 ± 1.14 and 3.18 ± 1.58, respectively. The VAS scores of both groups decreased compared with those before grouping, but there was no significant difference between the two groups (Fig. 4B). In contrast, in the non-AIT group, the VAS scores for the HRV-positive (70) and HRV-negative (103) subgroups reached 4.74 ± 1.22 and 4.13 ± 1.39, respectively, which were significantly different (*P* = 0.007), as demonstrated by an increase in the number of HRV-positive group scores of ≥6 (34% vs 15%) and a decrease in the number of mild symptoms (score 1–2, 1.4% vs 14%) (Fig. 4C). We further divided HRV-positive group into HRV-A, HRV-B, and HRV-C subgroups and evaluated the relationship between infection of different viral species and the severity of symptoms in non-AIT subjects. The data indicate that the VAS scores of viral negative group (4.13 ± 1.39) are significantly lower than those of the HRV-A (4.67 ± 1.27, *P* = 0.037)-, HRV-B (4.70 ± 1.06, *P* = 0.033)-, and HRV-C (4.91 ± 1.35, *P* = 0.015)-positive groups (Fig. 4D). No significant differences of VAS scores were found between A and B, B and C, and A and C groups (*P* = 0.944, 0.888, and 0.707) (Fig. 4D). Together, these results suggest that URT-HRV positivity (regardless viral species) is associated with the severity of symptoms in AR children who are not treated for allergies.

**Fig 5:**
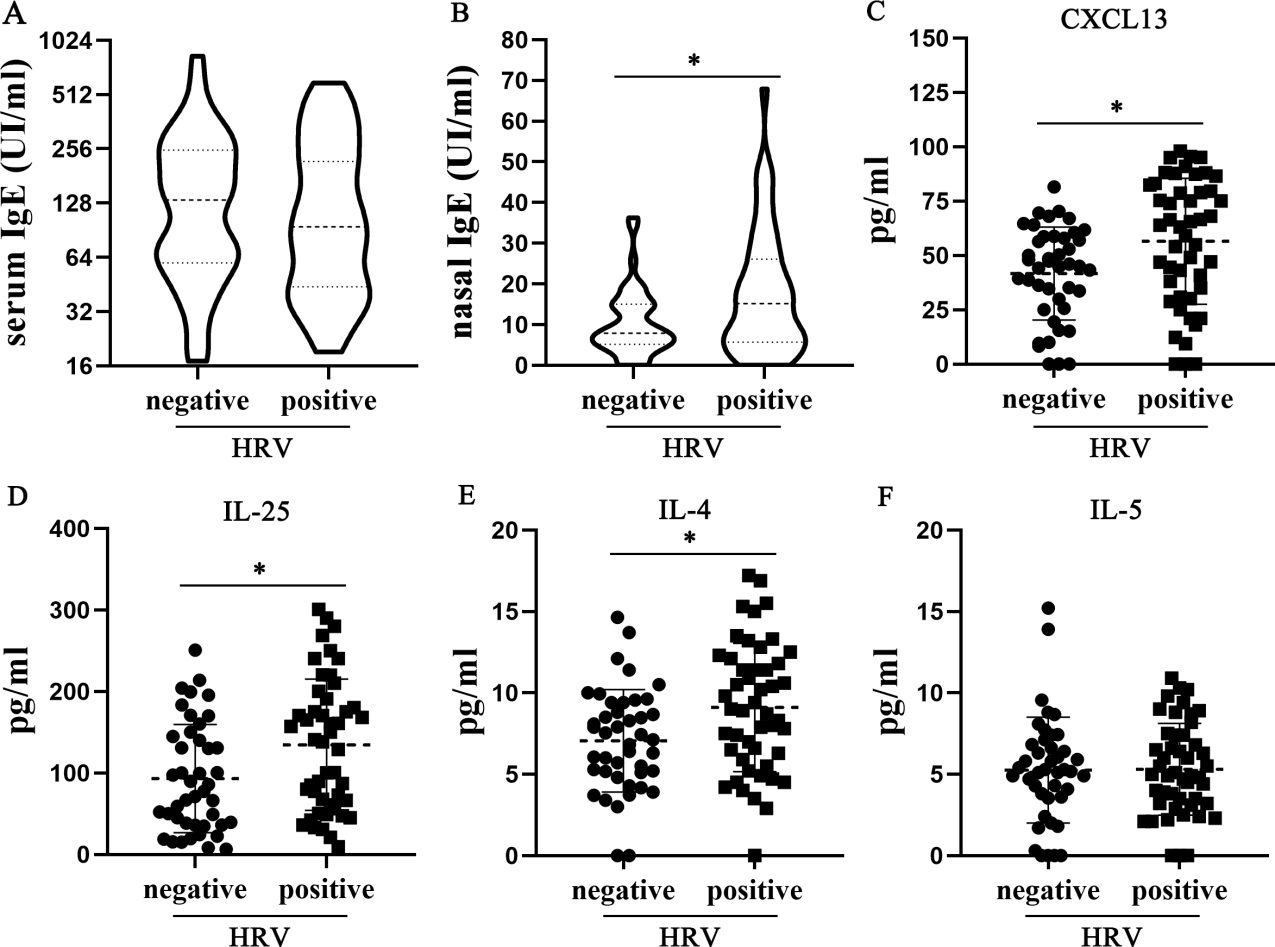
IgE and inflammatory cytokine levels between HRV-positive and HRV-negative groups in non-AIT AR subjects. (**A**) Total serum IgE levels in HRV-positive group (*n* = 54) vs HRV-negative group (*n* = 49). (**B**) Total nasal lavage IgE levels in HRV-positive group (*n* = 54) vs HRV-negative group (*n* = 49). (**C–F**) Nasal lavage levels of CXCL13, IL-25, IL-4, and IL-5 cytokines in HRV-positive group (*n* = 54) vs HRV-negative group (*n* = 49). Data are presented as the mean ± SD. ^*^P < 0.05 based on two-tailed Mann–Whitney U-tests (**A and B**) and two-tailed *t*-test (**C–F**).

To further identify the correlation between HRV detection and allergic symptoms, system and local IgE levels were measured between HRV-positive and HRV-negative groups in non-AIT subjects. Although the serum IgE levels were similar between the two groups (Fig. 5A), the nasal IgE levels in the HRV-positive group (17.52 ± 15.30) were significantly higher (*P* = 0.007) than those in the negative group (10.29 ± 8.28) (Fig. 5B), indicating that HRV infection may affect local allergic responses. Airway inflammation in AR is a type 2 immune response mediated by key effector cytokines like IL-4, IL-5, and IL-13, as well as upstream “alarmins” such as IL-25, IL-33, and thymic stromal lymphopoietin (15). The measurements of nasal IL-25, IL4, and IL-5 levels in the HRV-positive and HRV-negative groups of non-AIT subjects showed that the positive group had higher levels of IL-25 (*P* = 0.009) and IL-4 (*P* = 0.008), while IL-5 levels were comparable between the two groups (Fig. 5D through F). Miniscule levels of IL-33 and IL-13 were detected in nasal lavage fluid. Moreover, the levels of nasal B-cell chemokine CXCL13 in the HRV-positive group (56.61 ± 28.99) were significantly higher than in the negative group (41.71 ± 21.36) (*P* = 0.007) (Fig. 5C). The above data suggest that HRV infection contributes to the development of local type 2 immune responses in AR children.

### Isolation of HRV strains and phylogenetic and mutation analysis

**Fig 6:**
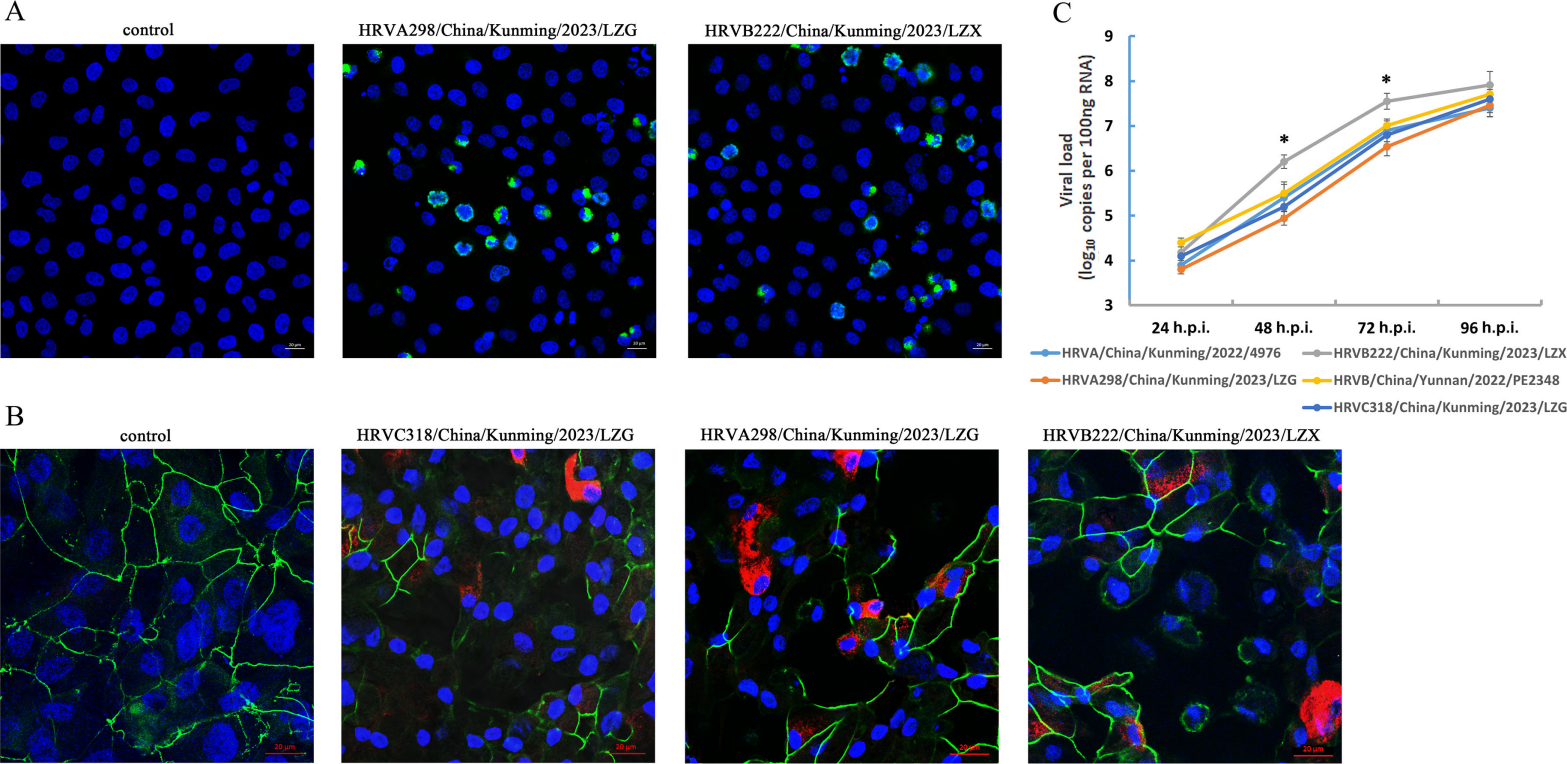
Viral isolates *in vitro* infections. (**A**) Immunofluorescence detection of HRV VP3 viral protein (green) and cell nuclei (blue) at 48 hours post-infection (h.p.i.) of HRVA298/China/Kunming/2023/LZG and HRVB222/China/Kunming/2023/LZX strains in H1-HeLa-infected cells by confocal microscopy. (**B**) Immunofluorescence detection of HRV VP3 viral protein (red), epithelial tight junction marker ZO-1 protein (green), and cell nuclei (blue) at 72 h.p.i. with the HRVC318/China/Kunming/2023/LZG, HRVA298/China/Kunming/2023/LZG, and HRVB222/China/Kunming/2023/LZX strains in ALI-NECs by confocal microscopy. (**C**) Viral loads were assessed based on the number of VP2 gene RNA copies in the infected ALI-NECs at different h.p.i. by quantitative real-time PCR (qRT-PCR). The error bars represent the SD from four repeats. ^*^P < 0.05 based on two-tailed *t*-test.

To further characterize HRV in children with AR, we carried out *in vitro* isolation of HRV strains (species A and B) from clinical samples using H1-Hela cells. HRV-C cannot be effectively propagated in immortalized cell lines (such as H1-HeLa) but can be amplified in *in vitro* sinus organ culture and ALI-cultured differentiated human airway epithelial cells due to its unique host receptor human cadherin-related family member 3 (16–18). We therefore built an ALI-cultured primary human airway epithelial organoid model to isolate species C from the positive sample. Among the 101 positive samples, four samples showed obvious CPE after incubation with H1-HeLa and viral VP3 protein that were detected in the infected cells (Fig. 6A). Viruses from the four samples were further propagated, and viral RNA was extracted for whole-genome sequencing. Two HRV-A strains, HRVA/China/Kunming/2022/4976 (GenBank No. OQ747182) and HRVA298/China/Kunming/2023/LZG (GenBank No. OR220809), and two HRV-B strains, HRVB222/China/Kunming/2023/LZX (GenBank No. OQ533275) and HRVB/China/Yunnan/2022/PE2348 (GenBank No. OQ747183), were identified. One sample of HRV-C was inoculated in ALI-cultured nasal epithelial cells (ALI-NECs), and expression of viral VP3 protein and disruption of the epithelia tight junctions near the VP3 detection area were observed in the infected ALI-NECs (Fig. 6B). The whole genome of the propagated virus was sequenced and identified as an HRV-C strain HRVC318/China/Kunming/2023/LZG (GenBank No. OR220808). All isolated strains of different species were able to infect and proliferate on ALI-NECs, with the HRVB222 strain having a faster proliferation kinetics compared to other virus strains (Fig. 6B and C).

**Fig 7:**
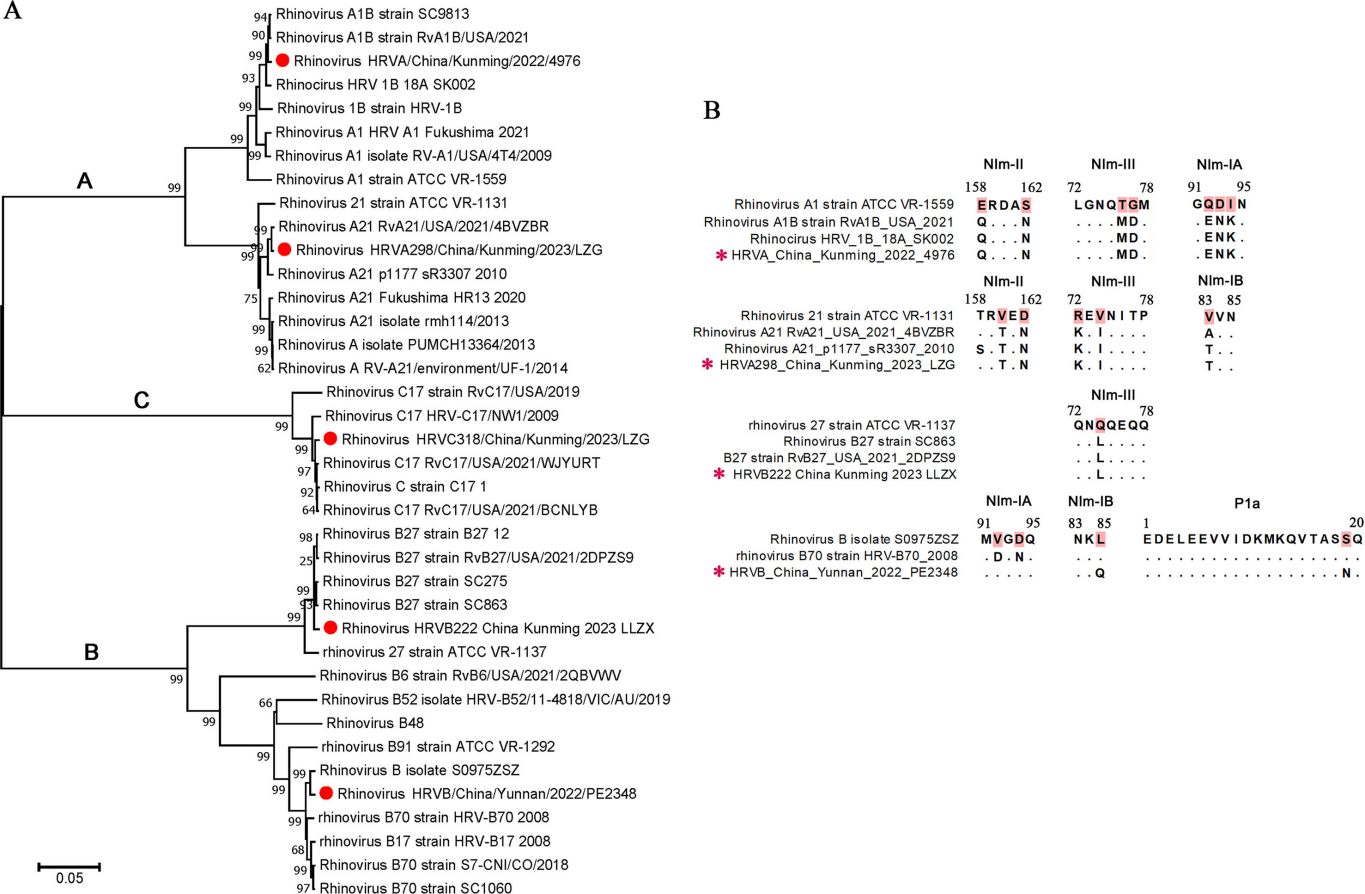
Phylogenetic and variation analysis of the isolated HRV strains. (**A**) Phylogenetic analysis of the complete genome sequence of the five isolated HRV strains and reference HRV gene sequences from the GenBank database. The five isolated HRV strains are labeled with red circles. (**B**) Amino acid variation analysis by multiple sequence alignment of the NIm-IA, NIm-IB, NIm-II, NIm-III, and P1a antigen epitopes in VP1 (NIm-IA, NIm-IB, and P1a), VP2 (NIm-II), and VP3 (NIm-III) proteins of the isolated HRV strains together with the homologous reference strains in Fig. 4A. Variable amino acids are highlighted in red in the reference sequences. The black dot indicates the same amino acid as the reference sequence. The isolated strains are labeled with red stars.

A phylogenetic tree was constructed based on the complete sequences of the five isolated HRV strains together with the homologous HRV reference sequences from GenBank, which were divided into three major branches of species A, B, and C (Fig. 7A). The two isolated A strains are A1B and A21, with amino acid sequence consistencies of up to 97.80% and 97.83% with neighboring virus strains, respectively. The two isolated B strains are B27 and B70, with amino acid sequence consistencies of up to 97.80% and 95.52% with neighboring virus strains, respectively. The isolated type C strain was C17 (97.72% amino acid sequence identity of neighboring virus strain RvC17/USA/2021/WJYURT). As a single-stranded RNA virus, HRV homologous recombination and sequence mutation are the causes of antigen drift and new genotype generation. SimPlot software analysis showed that there was no homologous recombination between the five isolates and their homologous reference strains (data not shown). Viral capsid proteins VP1, VP2, and VP3 are the key viral molecules in viral attachment, determining the antigenic epitopes and neutralizing antibody epitopes of HRV. We analyzed the amino acid variation of four neutralizing antibody epitopes (NIm-IA, NIm-IB, NIm-II, and NIm-III) of viral VP1-3 proteins and the P1a sequence of VP1 which is related to immune escape from rhinovirus infection (19–21). The A1B, A21, and B27 isolates had the same sequence of the four neutralizing antibody epitopes as their homologous reference strains but exhibited varying degrees of mutation compared to that in the ATCC prototype strains (Fig. 7B). The B70 isolate had variations in D92V and N94D of the NIm-IA epitope compared with the homologous reference strain HRV-B70_2008 and in L85Q of the NIm-IA epitope and S19N of the P1a sequence compared with HRV-B70_2008 and S0975ZSZ reference strains (Fig. 7B).

## DISCUSSION

HRV causes acute respiratory infections and influences the development of chronic airway diseases in humans worldwide (6). Most current epidemiological analyses of respiratory HRV infection have focused on URT influenza-like cases and rhinosinusitis, as well as LRT community-acquired pneumonia (CAP), COPD, and acute asthma attack or exacerbation (6, 22), while little is known about the etiology of HRV in AR. In this study, the detection rate of URT-HRV in AR children in Southwestern China reached 40%. The detection rate is higher than the URT-HRV detection rate in asymptomatic infants and children (~14%) (23, 24), and there was no local HRV epidemic between 2022 and 2023, based on surveillance data from Yunnan Center for Disease Control and Prevention, indicating a strong correlation between HRV infection and childhood AR. Meanwhile, the detection rate is also higher than the positive rates of HRV in Chinese children with acute respiratory tract infection (17.4%) (25) and in children with CAP (12.2%–29%) (26–28), further revealing the high prevalence of HRV in the URT of AR children. A survey of 58 adult patients with perennial AR detected respiratory viruses in the nasal cavity of 43.1% of the patients, which was significantly higher than that of normal controls, and more than half of the detected viruses were HRV (9). Our study found an even higher detection rate of HRV in AR children than in adults (22.4%) (9), which is probably owing to the increasing number of children with AR and the susceptibility of children to HRV (2, 3, 6). The peak detection period of HRV in AR children in our study is in the autumn, which is possible due to the seasonality of HRV (22). The high infection rate suggests that the allergic environment in the URT of AR children may promote HRV infection. Indeed, allergic inflammation disrupts the airway epithelial barrier and upregulates the expression of the HRV receptor ICAM-1 in the airway, contributing to HRV infection (29). HRV infection itself can upregulate ICAM-1 receptor expression on NECs too (30). Studies have also found that atopic bronchial epithelial cells have reduced antiviral type 1 interferon function and thus increased HRV infection (31). Furthermore, we found that the prevalence of HRV in AR children was mainly species A and B (accounting for nearly 80%), which differs from that of the majority of species A and C in acute or severe respiratory infection detections (6, 22). The immune debt caused by social isolation of COVID-19 does not seem to lead to the change of HRV epidemic diversity, as the survey of patients with acute respiratory infection/influenza-like illness after COVID-19 (including China) shows that the main epidemic strains of HRV are still A and C species (32–35). In overall respiratory infectious diseases, the majority of HRV genotypes detected are the A12, A78, and C2 (22). However, in AR, B27, C2, and B70 are the top three HRV genotypes in this study in terms of detection rates. It is interesting that the high frequency of HRV-B appears in AR children and is related to the severity of symptoms, considering that HRV-B is more frequently associated with asymptomatic subjects and less likely to cause severe respiratory illnesses and exacerbations of asthma. Perhaps HRV-B is more adaptive to the URT environment, while HRV-A and HRV-C are more likely to invade the LRT and cause serious respiratory diseases than HRV-B. Our results found that the isolated B27 strain has a more efficient *in vitro* proliferation on primary NECs compared to other isolated strains (Fig. 6C); the B70 strain acquires extra mutations at the P1a site related to immune escape (Fig. 7B), indicating that the two HRV-B strains isolated from AR children in our study are evolutionarily more adaptive to host URT infections. The relationship between HRV-B infection and AR needs further exploration. Nevertheless, these data suggest distinct species prevalence characteristics of HRV in URT allergic diseases compared to other HRV infection respiratory diseases.

Our study revealed a correlation between HRV detection and symptom severity in AR children who did not receive allergy-specific treatment, with HRV-positive AR children having more severe allergic symptoms. Higher local nasal IgE, IL-4, and IL-25 levels presented in HRV-positive subjects suggest a role of HRV infection in contributing type 2 immune/inflammation responses of the AR children. HRV infection can cause the release of pro-inflammatory factor IL-25 from respiratory epithelial cells, further promoting pulmonary production of type 2 immune/inflammation effectors IL-4, IL-5, IL-13, and IgE, to exacerbate asthma allergic responses (36). Besides, we found increasing CXCL13 expression in the nasal lavage of HRV-positive subjects. CXCL13 is a B-cell chemoattractant and plays a key role in the formation of nasal-associated lymphoid tissue (NALT) (37). As a tertiary lymphoid tissue, NALT contributes to local B-cell activation, survival, and antibody production in chronic rhinosinusitis (38). Considering the increasing level of local IL-4 which is responsible for IgE class transition in B-cell, it is possible that HRV infection induces *de novo* local IgE production by NALT in allergic nasal tissues. Whether URT-HRV infection can induce nasal IgE production of AR patients and therefore exacerbates symptoms needs further investigation.

Finally, we isolated HRV strains involving all three HRV species from nasal lavage fluid samples of AR children. These strains efficiently infected primary human NECs *in vitro*, suggesting that the URT of AR children is a natural reservoir of HRV and has transmission potential to healthy populations. In addition, according to the variation in the antigenic and neutralizing epitopes of VP1-3 proteins of the isolated viral strains compared to homologous viral strains, HRV exhibits progressive mutation/evolution in AR pediatric hosts. It is necessary to strengthen the monitoring and intervention of children’s respiratory HRV infection.

## Data Availability

The data that support the findings of this study are available from the corresponding author upon reasonable request. Complete genome sequences of HRVs obtained in this study were submitted to the GenBank database under the accession numbers OQ533275, OQ747182, OQ747183, OR220808, and OR220809.
